# Cost-Effectiveness of Infant Pneumococcal Conjugate Vaccination Strategies in Vietnam: A Stepwise Economic Evaluation

**DOI:** 10.3390/vaccines14030220

**Published:** 2026-02-27

**Authors:** Liping Huang, An Ta, Artem Antonov, Michael Groff, Phong Lan Nguyen

**Affiliations:** 1 Health Economics and Outcomes Research, Pfizer Inc., Collegeville, PA 19426, USA; 2 Cytel, Hamilton House, Mabledon Place, London WC1H 9BB, UK; an.ta@cytel.com; 3 Cytel, 3012 NJ Rotterdam, The Netherlands; artem.antonov@cytel.com; 4 Cytel, Vancouver, BC V5Z 1C6, Canada; michael.groff@cytel.com; 5 Pfizer (Vietnam) Ltd., Thành phố Hồ Chí Minh 700000, Vietnam

**Keywords:** pneumococcal conjugate vaccine, PCV, pneumococcal disease, invasive pneumococcal disease, pneumonia, otitis media

## Abstract

**Background:** Vietnam is one of few remaining countries without a pediatric pneumococcal National Immunization Program (NIP). However, four pneumococcal conjugate vaccines (PCVs) are available in Vietnam: 10-, 13-, 15-, and 20-valent PCVs (PCV10, PCV13, PCV15 and PCV20). Given the availability of multiple PCVs, selecting an optimal vaccination strategy is challenging. This paper aims to estimate the vaccination impact of these PCVs, with and without the implementation of a pediatric NIP, to inform decision-makers and healthcare providers. **Methods:** A Markov model was adapted to evaluate the impact of all vaccines administered under a 3 + 1 schedule (50% vaccine uptake with direct protection assumed only) and a hypothetical scenario including PCVs 2 + 1 in Vietnam’s pediatric NIP (90% uptake with both direct and indirect protection) from a payer’s perspective. For each scenario, we performed stepwise comparisons of each vaccine with the next higher-valent option: PCV13 versus PCV10, PCV15 versus PCV13, and PCV20 versus PCV15. **Results:** Under the 3 + 1 schedule, PCV13 and PCV20 were cost-effective versus PCV10 and PCV15, respectively. PCV15, however, was not cost-effective versus PCV13, though offering greater health benefit but at a higher total cost. Under the 2 + 1 schedule, PCV13 remained cost-effective over PCV10, while PCV15 was not cost-effective relative to PCV13. PCV20 was dominant over PCV15. Sensitivity analyses demonstrated results consistent with both reference cases. **Conclusions:** Vaccinating infants in Vietnam through the private market or an NIP with PCV13 or PCV20 was estimated to be more cost-effective or cost saving than strategies based on PCV10 or PCV15, respectively. These findings provide valuable evidence to inform policy decisions.

## 1. Introduction

Pneumococcal diseases include invasive pneumococcal disease (IPD), which mainly represents as meningitis, a severe infection of the membranes surrounding the brain and spinal cord that can lead to life-threatening neurological complications, and bacteremia, which is a bloodstream infection that may rapidly progress to sepsis. Pneumococcal diseases also include non-invasive manifestations such as pneumonia, a lung infection characterized by fever, cough, and respiratory distress, and acute otitis media (OM), which is a middle-ear infection causing pain, fever, and fluid accumulation [1]. Although *Streptococcus pneumoniae* (*S. pneumoniae*) can cause other clinical syndromes, these conditions represent the most prevalent and clinically significant manifestations in children and are prominent causes of childhood morbidity and mortality globally [2].

As this paper focused on children under 2 years of age, polysaccharide pneumococcal vaccines are not considered because they are not indicated for this age group due to limited immunogenicity. Therefore, this analysis evaluates pneumococcal conjugate vaccines (PCVs), which target multiple *S. pneumoniae* serotypes (i.e., the leading cause of bacterial pneumonia worldwide) and have been developed and launched in many countries across the globe [3]. The introduction of PCVs has had a considerable impact on the global burden of pneumococcal disease with an estimated 175.2 million pneumococcal disease cases and 0.6 million deaths prevented by PCV13 between 2010 and 2019 [4].

In Vietnam, pneumonia is the primary cause of mortality among children younger than 5 years with a reported 2.9 million cases and 4000 deaths annually [5]. Precise national estimates are challenging due to the absence of routine nationwide pneumococcal surveillance, as most available data come from passive reporting and small, ad hoc studies; nevertheless, these studies consistently show that young children bear the highest burden—84.7% of laboratory-confirmed IPD cases occur in children under 5 years of age [6,7]. There are currently several pneumococcal vaccines marketed in Vietnam that cover multiple, incremental *S. pneumoniae* serotypes: the 10-valent PCV (PCV10; covering *S. pneumoniae* serotypes 1, 4, 5, 6B, 7F, 9V, 14, 18C, 19F, and 23F), the 13-valent PCV (PCV13; covering PCV10 serotypes and 3, 6A, and 19A), the 15-valent PCV (PCV15; covering PCV13 serotypes and 22F and 33F), and the 20-valent PCV (PCV20; covering PCV15 serotypes and 8, 10A, 11A, 12F, 15B). In Vietnam, both PCV10 and PCV13 have been licensed and available in the private market for children since 2013 and 2018, respectively [8], while PCV15 and PCV20 were introduced as an option for infant and young children in May and November 2025, respectively [9,10].

Despite the availability of higher-valent options, vaccine-preventable serotypes dominate pneumococcal disease in Vietnam [6]. The most common IPD serotypes identified between 2019 and 2022 were 6A/B, 19A, 23F, 19F, and 14, corresponding to an estimated coverage of 66.0% for PCV10/PCV7, 83.0% for PCV13, and 87.2% for PCV20 [6]. These patterns align with nasopharyngeal carriage studies involving more than 13,000 Vietnamese children where vaccine serotypes were similarly prevalent among healthy carriers. High levels of antimicrobial resistance—particularly macrolide resistance approaching 100%, and ceftriaxone and cefotaxime nonsusceptibility exceeding 70% in IPD isolates—further underscore the importance of prevention through vaccination [7].

As Vietnam prepares to introduce a PCV into its NIP in the near future, evidence-informed vaccine selection will be essential [7]. Countries that have implemented PCVs at the national level have achieved large and sustained reductions in pneumococcal disease cases, deaths, and antimicrobial resistance, which have been driven by both direct protection in vaccinated children and substantial herd effects across all age groups [7]. Given Vietnam’s current serotype landscape, documented disease burden, and high antimicrobial resistance, robust local evidence will play a critical role in guiding NIP product choice and maximizing the future public health impact of PCV introduction.

To date, evidence on the economic value of PCVs, both in pediatric and adult population, in Vietnam remains limited. While a conference abstract by Nguyen et al. 2024 [11] compared the cost-effectiveness of PCV13 and PCV10 in Vietnamese children, this analysis was not peer-reviewed. Notably, no published economic evaluations or public health impact models were identified for higher-valent PCVs in the Vietnamese pediatric population.

In this context, a stepwise cost-effectiveness analysis was conducted to evaluate the potential health and economic impact of vaccinating infants in Vietnam with PCV20, PCV15, PCV13, or PCV10, comparing each higher-valent option against the next most relevant alternative (i.e., rather than a direct comparison with “no vaccine”) in terms of their relative impact on the population under different sets of assumptions. This approach reflects how decision-makers would incrementally assess whether each change in intervention value justifies the additional cost and aims to guide healthcare decision-making regarding the optimal PCV vaccination strategy.

## 2. Methods

### 2.1. Model Structure

A multi-cohort decision-analytic Markov (state-transition) model developed in Microsoft Excel^®^ (Redmond, WA, USA) using a combination of standard Excel formulas and custom Visual Basic Application (VBA) scripts was adopted to assess the cost-effectiveness of vaccinating infants in Vietnam with PCV20, PCV15, PCV13, or PCV10 with a no-vaccination option as a standard of care (SoC). The model structure has been widely accepted and commonly used in the assessment of costs and health benefits associated with pneumococcal vaccines [12,13,14,15,16,17].

The modelling horizon was 10 years, corresponding to ten successive birth cohorts. However, health outcomes including life years (LYs) and quality-adjusted life years (QALYs) were assessed over the individual’s lifetime. When deaths occurred during the model period leading to the loss of LYs and QALYs, such remaining expected health outcomes were accumulated over the individual’s remaining lifespan and discounted from the year of death. A ten-year horizon was deemed sufficient to capture the maximum direct and indirect effects of infant pneumococcal vaccination while minimizing the unnecessary uncertainty associated with longer projections. Evidence from multiple settings indicates that herd protection in older age groups emerges within the first few years following vaccine introduction and subsequently stabilizes [18].

**Figure 1:**
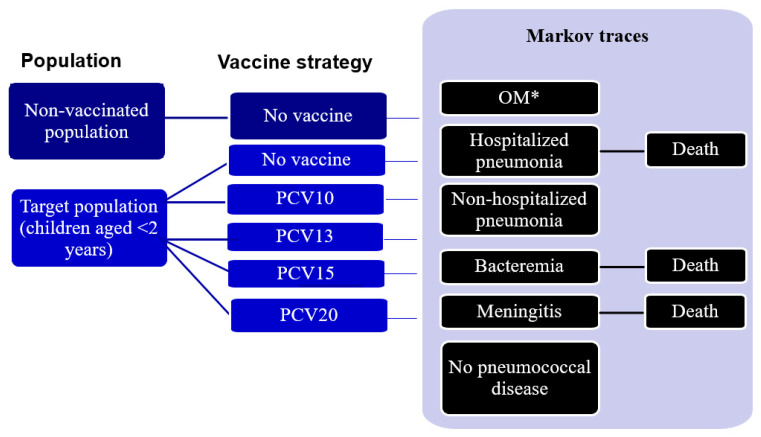
Model diagram. The probability of death considered general mortality and case fatalities. * Pediatric population (<18 years old) only. Abbreviations: OM, otitis media; PCV10, 10-valent pneumococcal conjugate vaccine; PCV13, 13-valent pneumococcal conjugate vaccine; PCV15, 15-valent pneumococcal conjugate vaccine; PCV20, 20-valent pneumococcal conjugate vaccine.

Costs and health benefits in the model were discounted at a rate of 3% per annum in line with a previously published cost-effectiveness analysis in Vietnam [19]. In annual cycles, within the disease active state, vaccinated and unvaccinated individuals could experience several clinical events, including IPD (delineated as meningitis or bacteremia) with the possibility of resulting death; all-cause pneumonia (either non-hospitalized or hospitalized with the possibility of resulting death); all-cause OM (only for the pediatric population < 18 years old); or remain in a “no pneumococcal disease” state (Figure 1). Clinical events within the pneumococcal disease states were modeled as non-mutually exclusive, allowing individuals to experience one or more clinical events in each yearly cycle. The probability of death was considered as a composite of general mortality and case fatalities for meningitis, bacteremia, or hospitalized pneumonia. Annual variations in transition probabilities for individual health states considered age and vaccination status.

The model incorporated multiple cohorts to simulate temporal dynamics with a proportion of a new birth cohort entering the model and being vaccinated at the commencement of each annual cycle. The present study tested two sets of assumptions (reference cases) to reflect alternative policy pathways relevant for Vietnam. The first reference case represented the current situation in which PCVs are not included in the NIP, and vaccines are optional through the private market. The second reference case reflected a potential future scenario in which PCVs are introduced into the NIP. In the first reference case, moderate vaccine uptake was assumed (i.e., 50% of infants and young children would receive vaccination). Given the relatively small vaccine coverage, the indirect effect in the existing population (i.e., assuming no herd immunity) was not modeled. With PCV not currently being a part of a pediatric NIP in Vietnam, access to vaccination is mainly available through private vaccination centers in which a 3-dose primary series with a booster (i.e., a 3 + 1 schedule) has been approved by the Ministry of Health (MoH). Therefore, in the first reference case, all four vaccines were assumed to follow a 3 + 1 schedule of immunization, and indirect protection was not assumed. The study population included a vaccinated cohort consisting of vaccinated children (aged < 2 years) and an unvaccinated cohort (i.e., SoC) comprising children aged ≤ 17 years. The modeled populations were stratified by age group: <1 year, 1 year, 2 years, 3 years, 4 years, and 5 to 17 years. The second reference case modeled a hypothetical scenario considering the inclusion of a PCV into the pediatric NIP in Vietnam and aimed to identify the most efficient vaccine and to evaluate its cost-effectiveness among all four vaccines (i.e., PCV20, PVC15, PCV13, and PCV10). A 90% vaccine coverage was assumed for the full course of vaccination under a 2 + 1 schedule, which is consistent with coverage levels observed in neighboring countries with PCV included in the NIP. Countries such as Malaysia [20,21], Singapore [22], and Taiwan [23] implemented a 2 + 1 PCV schedule in their NIPs and have demonstrated high vaccine uptake through their infant immunization program. On this basis, in the second reference case, indirect protection was considered, which means that the non-vaccinated individuals were assumed to benefit from the indirect effect (i.e., herd immunity). Therefore, the model population included the entire Vietnamese population to comprehensively assess the impact of the pediatric vaccination program in the country. The indirect effect associated with each vaccine was assumed to increase progressively until the fifth year following the implementation, reaching its maximum, and then remain stable at this level for the duration of the modeling period. The model stratified the pediatric population as described in reference case 1, while the adult population was split into the following groups: 18 to 34 years, 35 to 49 years, 50 to 64 years and ≥65 years. Assumptions for each reference case are summarized in Supplementary Table S1.

### 2.2. Model Inputs

The inputs used in this study were exclusively derived from secondary analyses of previously published, aggregated, and non-identifiable data. Therefore, institutional review board approval and informed consent were not required. The key model inputs are listed in Table 1, Table 2 and Table 3; where possible, the data from Vietnam were prioritized in the model.

Direct vaccine effects against non-invasive diseases were adjusted using serotype coverage pre-PCV7 to pre-era for higher-valent vaccines. PCV7 all-cause efficacy data were adjusted from pre-PCV7 era (80.6% PCV7 serotype coverage) to the current level of serotype coverage observed in the Vietnamese population for every PCV. Direct effect data for all the vaccines on vaccine-type IPD were based on observed PCV13 vaccine effectiveness data sourced from Savulescu et al., 2022 [36] (assumed at 88.7% for all vaccine serotypes regardless of vaccine) and adjusted for currently observed serotype coverage in the Vietnamese population.

Population size by sex and age was extracted from the World Bank population estimations and projections. In 2025, Vietnam’s total mid-year population was projected to be 101,598,529, including 27.8 million of children aged <18 years, and an adult population (≥18 years) of almost 74 million (Table 1) [24]. The annual new birth cohort was derived from the World Bank population estimations and projections [24].

For the age groups under 5 years, IPD incidence rates were sourced from Anh et al., 2009 [25], which reported invasive pneumococcal-associated disease among hospitalized children aged under 5 years in Khanh Hoa Province, Vietnam, between April 2005 and August 2006 (Table 1).

Due to the lack of Vietnam-specific data, a Thai-population study by Dilokthornsakul et al., 2019 [26] was used to obtain the incidence rates of IPD in the ≥5 years age groups, the share of meningitis cases among all IPD, all-cause hospitalized and non-hospitalized pneumonia, and all-cause OM for all age groups (Table 1). Thailand has a comprehensive surveillance system and, similar to Vietnam, has not implemented a PCV in the NIP for children and adults. Therefore, Thai data were deemed a suitable proxy for this analysis.

The model did not account for sequelae resulting from pneumococcal disease due to insufficient robust Vietnamese data.

A targeted literature review was conducted to find the serotype distribution of IPD, from which six publications were identified [6,8,48,49,50,51] (Supplementary Table S2). Each study was conducted in one to three hospitals in Vietnam, focused on certain age groups, and included a relatively small sample size. Therefore, none of these studies could be considered nationally representative and fully reflective of *S. pneumoniae* serotype distribution in the general population in Vietnam. To estimate serotype coverage for each of the vaccines, the mean coverage was calculated from five out of the six above-mentioned studies. The study by Lien et al., 2023 [51], was excluded from the calculations, as it did not differentiate between serotypes 6A and 6B (i.e., 6B is covered by all the vaccines considered, while 6A is not covered by PCV10). Additionally, one meta-analysis was identified, which was excluded from the analysis as it did not consider serotypes exclusively covered by PCV15 and PCV20 [7].

Disease-associated cost data (i.e., medical cost per disease episode) was obtained from Anh et al., 2010 [29] for children aged under 5 years (cost data collected in 2006) and Vo et al., 2018 [30] for the population older than 5 years (cost data collected in 2016). The costs in these studies were originally reported in United States dollars (USD). They were first converted to Vietnamese dong (VND) using the exchange rates at the time that the respective studies were conducted and then inflated to 2025 prices using yearly consumer prices indices [31,32]. The inflated VND costs were then reconverted to USD using the exchange rate of 25,950 VND/USD (median of daily exchange rate of 2025 through August 20 [52]) (Table 1).

The vaccination cost used for this analyses was based on the published vaccine list price [53], in which the cost per dose for PCV13, PCV15 and PCV20 was 23%, 57% and 66% more than that for PCV10, respectively, and the vaccine administration costs were not considered.

The age-specific utility for the general healthy population was informed by Nguyen et al. 2017 [42]. QALY decrements for IPD (meningitis and bacteremia), pneumonia (hospitalized and non-hospitalized), and OM were sourced from the published literature and applied to the corresponding age groups (Table 3) [43,44,45,46,47].

For the assumptions about the direct and indirect effect of the vaccine, the estimation was based on published PCV13 effectiveness and impact studies as well as PCV7 clinical trials with selection based on study size, quality of data, and dosing schedule. In reference case 1, only direct vaccine effects were considered given the relatively low assumed vaccine uptake assumption (50% for all vaccines), as PCVs are currently not included in the NIP for infants and children in Vietnam. Direct effect data for all the vaccines on vaccine-type IPD were based on observed PCV13 vaccine effectiveness data sourced from Savulescu et al., 2022 (assumed at 88.7% for all vaccine serotypes regardless of vaccine) [36]. Considerable heterogeneity in study design and endpoint definition, as well as variable results, were observed in PCV13 effectiveness and impact studies for vaccine effect on non-invasive disease. Therefore, efficacy data from PCV7 pivotal trials were used to determine the direct effect on non-invasive disease. These were adjusted using the ratio between the current serotype coverage for each vaccine versus the serotype coverage level for PCV7 (80.6%) in 2000 (i.e., when the PCV7 pivotal trial was conducted) [33,34,35]. The effectiveness of PCV13 and PCV10 on all-cause non-invasive disease was estimated using point estimates of vaccine efficacy against inpatient non-bacteremic pneumonia (25.5%), all-cause ambulatory non-bacteremic pneumonia (6.0%), and all-cause OM (7.8%) from the PCV7 pivotal trial [33,34,35] (Table 2a).

In reference case 1, to reflect the 3 + 1 vaccine schedule in which the three primary doses are given in the first year of life, the direct effect against IPD and non-invasive IPD after the completion of the priming doses (3 doses) was assumed at 75% of the full direct effect of the four-dose schedule. A similar adjustment was made for the 2 + 1 schedule in reference case 2, where all vaccines were assumed to have 67% of their full effect against all the outcomes during the first year of a person’s life. Direct vaccine effects were assumed to decrease by 10% annually from year 6 post-vaccination, reaching 59.05% of its maximum in year 10, which represented the maximum protection duration of all vaccines.

For reference case 2, indirect effects (assuming PCVs included in the NIP at 90% uptake of the complete schedule) were estimated using the maximum reduction in disease incidence and the accrual of this secondary effect over time (i.e., referred to as “indirect effect accrual”). The maximum reduction in IPD from the indirect effect stratified by age groups was informed by surveillance data from Ladhani et al., 2018 [37]. Estimates for hospitalized and non-hospitalized pneumonia were sourced from PCV13 impact studies by Levy et al., 2017 [38] and Rodrigo et al., 2015 [39], while those for OM were informed by Lau et al., 2015 [40]. The indirect vaccine effect was applied for all the vaccines analyses respecting their serotypes prevalence in the population. The accrual of these effects was guided by the annual relative change in IPD incidence following the introduction of PCV13 compared with the incidence one year prior (Table 2b) [18,37].

### 2.3. Analyses

**Figure 2:**
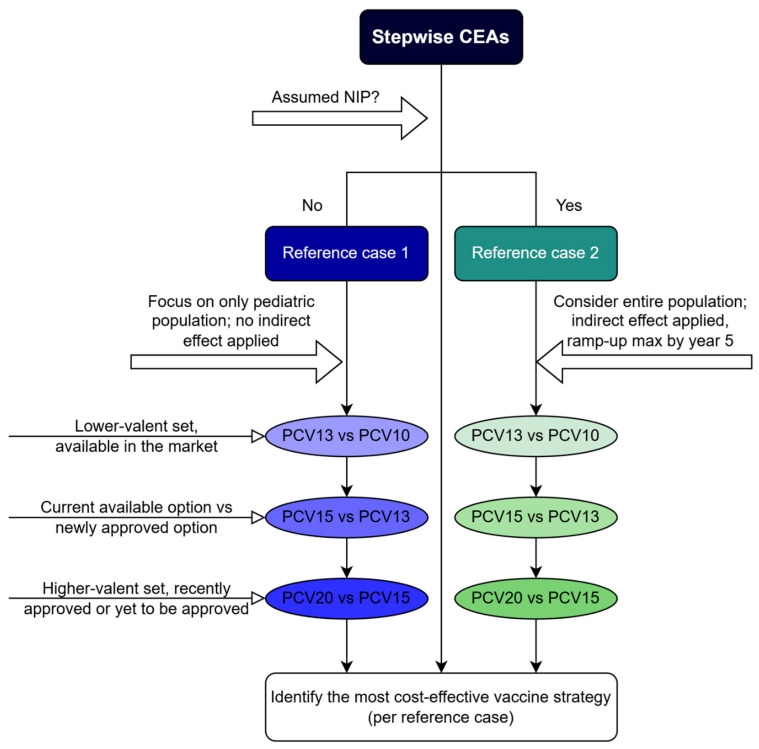
Stepwise analyses. Abbreviations: CEA, cost-effectiveness analysis; max, maximum; NIP, National Immunization Program; PCV10, 10-valent pneumococcal conjugate vaccine; PCV13, 13-valent pneumococcal conjugate vaccine; PCV15, 15-valent pneumococcal conjugate vaccine; PCV20, 20-valent pneumococcal conjugate vaccine.

In both reference cases, stepwise pairwise cost-effectiveness comparisons were conducted between PCVs that have been used in the private market (referred to as “lower-valent PCVs) such as PCV10 and PCV13 and the newly approved higher-valent PCVs including PCV15 and PCV20 in Vietnam. The analysis sequentially compared PCV10 with PCV13, followed by PCV13 versus PCV15, and finally PCV15 versus PCV20 to reflect the progressive introduction of higher-valent PCVs and to allow for an assessment of the incremental health gains and economic costs associated with each successive vaccine option. Such comparisons were intended to mirror how payers evaluate pneumococcal vaccine choices in practice as new products enter the market (Figure 2). In both reference cases, costs were accounted for from the payer’s perspective, accounting for direct medical costs only.

Each reference case was accompanied by both deterministic sensitivity analysis (DSA) and probabilistic sensitivity analysis (PSA) to assess the robustness of the results. In the DSA, each relevant parameter or group of parameters was varied individually at a default variance of 20% to estimate the upper and lower bounds, which were then used to compute incremental costs and incremental QALYs for each pairwise comparison. In the PSA, all relevant model parameters were randomly distributed with a default standard error of 10% over 1000 iterations. Vaccines costs per dose were not included in the PSA and DSA, as these parameters were sourced from the published list price and are not expected to change.

For each reference case, we additionally conducted a scenario analysis adopting a societal perspective, incorporating direct non-medical costs and indirect costs, to explore the broader economic impact of pneumococcal vaccination beyond the healthcare system. These components were estimated by applying the ratio of societal to direct medical costs reported by Le et al., 2014 [54] to the direct medical cost estimates used in the model. Direct non-medical costs included transportation expenses, costs incurred by household members during hospital visits, and miscellaneous expenditures such as lodging, soap, and diapers. Indirect costs reflected productivity losses experienced by caregivers.

The same direct non-medical and indirect costs were assumed for all IPD manifestations including meningitis and bacteremia. Societal costs were applied only to disease episodes requiring hospitalizations (bacteremia, meningitis, and hospitalized pneumonia) among children under 5 years of age. Societal costs were neither included for individuals aged 5 years and older, nor for non-hospitalized cases, due to the lack of reliable data for these groups.

### 2.4. Outcomes

Clinical outcomes included disease cases of IPD (meningitis and bacteremia), hospitalized and non-hospitalized pneumonia, and OM, plus deaths and QALYs. Assessed economic outcomes included direct medical costs due to pneumococcal disease, vaccination costs, and total costs. All outcomes were estimated separately for each vaccination option and the current SoC (i.e., no vaccination), and the differences between the two vaccination strategies (i.e., incremental results) were subsequently compared. Incremental cost-effectiveness ratios (ICERs) were calculated based on the incremental total cost divided by incremental QALYs for each pairwise comparison in both reference cases. As there is no recommended willingness-to-pay (WTP) threshold in Vietnam, two WTP options were considered in the analysis. In the first option, a threshold of three times the gross domestic product (GDP) per capita, as recommended by the World Health Organization [55,56] (i.e., projected GDP per capita of USD 4805.84 in 2025 [57], corresponding to a WTP threshold of USD 14,418) was used. The second option was more conservative and adopted based on Tran et al., 2025 [58], which estimated a mean WTP per QALY for a life-improvement scenario in Vietnam equivalent to 2.41 GDP per capita, yielding a WTP threshold of USD 11,582.10.

## 3. Results

### 3.1. Reference Case 1: Assuming No NIP

PCV13 was predicted to prevent 30,760 pneumococcal disease cases and 453 deaths over 10 years (Table 4). This reduction in disease translated into direct medical cost savings of almost USD 2.4 million and 20,071 QALYs versus PCV10. Since lower mortality under PCV13 resulted in more children entering later vaccination windows, the total number of vaccine doses administered was higher for PCV13 than for PCV10 along with a higher price per dose leading to a higher vaccination cost associated with PCV13. However, these additional vaccination costs were partially offset by reduced disease-related expenditure, yielding an ICER of USD 8077 per QALY. Using a conservative WTP threshold of USD 11,582.10 per QALY, PCV13 remained cost-effective compared with PCV10.

In the comparison of PCV13 and PCV15 (a recently approved option), the incremental gains for the higher-valent vaccine were limited due to the low prevalence (0.2%) of the two additional PCV15 serotypes in Vietnam. Although PCV15 was estimated to be more effective than PCV13, it was also considerably more costly due to substantially higher vaccination costs (i.e., the price per dose was almost 30% higher than PCV13). This resulted in an ICER of USD 1,069,181, indicating that PCV15 is not a cost-effective alternative to PCV13 under current epidemiological and cost assumptions.

In the comparison of the higher-valent vaccines that were recently approved in Vietnam (i.e., PCV20 and PCV15), PCV20 was estimated to prevent 13,587 cases of pneumococcal disease and 200 deaths over 10 years versus PCV15. This resulted in direct medical cost savings of more than USD 1 million and 8865 QALYs gained (Table 4). Although total vaccination costs were higher for PCV20 due to the increased numbers of doses purchased and higher costs per dose, reductions in disease burden almost completely compensated for these costs, resulting in an ICER of USD 285 per QALY. Under the conservative WTP threshold, PCV20 was a highly cost-effective option among high-valent vaccines.

The non-comparative results estimated for each vaccination strategy are in Supplementary Table S4.

### 3.2. Reference Case 2: Assumed NIP with PCV in Vietnam’s Pediatric Population

Evaluating potential vaccines for inclusion in the NIP required sequential comparisons (PCV10→PCV13→PCV15→PCV20) (Table 5) with non-comparative results presented in Supplementary Table S5. In these comparisons, ICERs generally increased with higher-valent vaccines except for the PCV20 versus PCV15 comparison, where PCV20 was shown to be dominant option, as it was more effective and less costly.

Overall, the pairwise comparison results in reference case 2 were broadly consistent with those observed in reference case 1. PCV13 remained a highly cost-effective option among the vaccination strategies currently used in Vietnam’s private market, while PCV20 was dominant in comparison with PCV15, and they were both driven by their broader protection against additional serotypes compared with their respective alternatives. In contrast, PCV15 was associated with better health gains than PCV13, which were accompanied by substantially higher vaccination costs, making it less favorable with an ICER of USD 117,292, far exceeding both WTP thresholds considered in this study. The efficiency frontier for both reference cases is presented in Supplementary Figures S1 and S2.

### 3.3. Sensitivity Analyses

**Figure 3:**
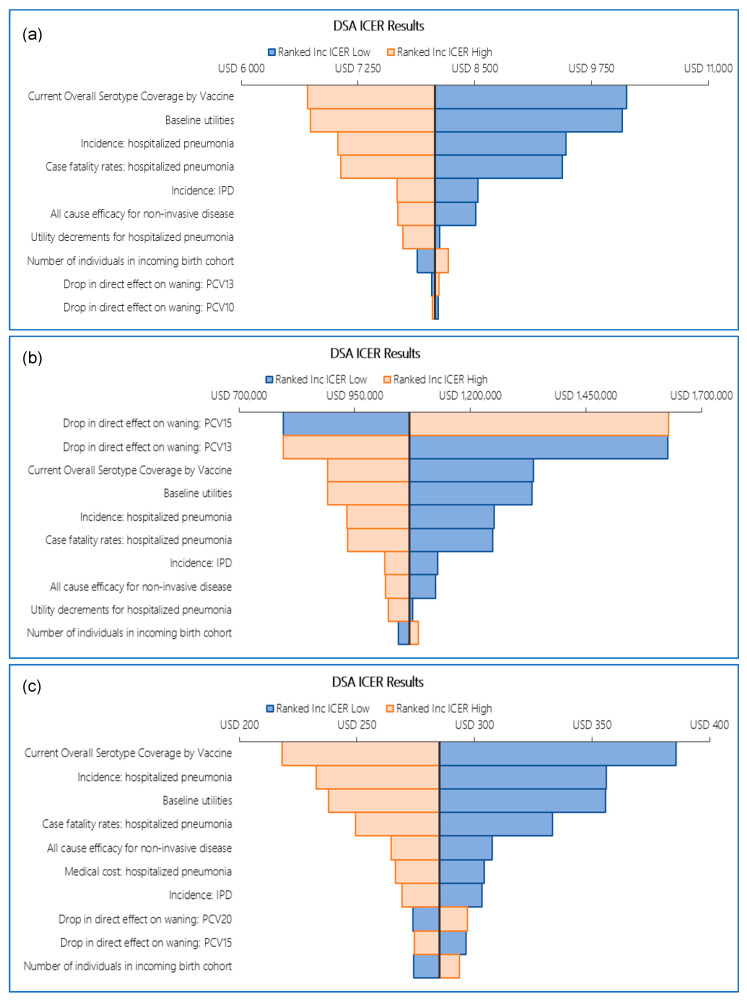
DSA ICER tornado diagram reference case 1: (**a**) PCV13 vs. PCV10, (**b**) PCV15 vs. PCV13, (**c**) PCV20 vs. PCV15. Abbreviations: DSA, deterministic sensitivity analysis; ICER, incremental cost-effectiveness ratio; inc, incremental; IPD, invasive pneumococcal disease; PCV10, 10-valent pneumococcal conjugate vaccine; PCV13, 13-valent pneumococcal conjugate vaccine; PCV15, 15-valent pneumococcal conjugate vaccine; PCV20, 20-valent pneumococcal conjugate vaccine.

**Figure 4:**
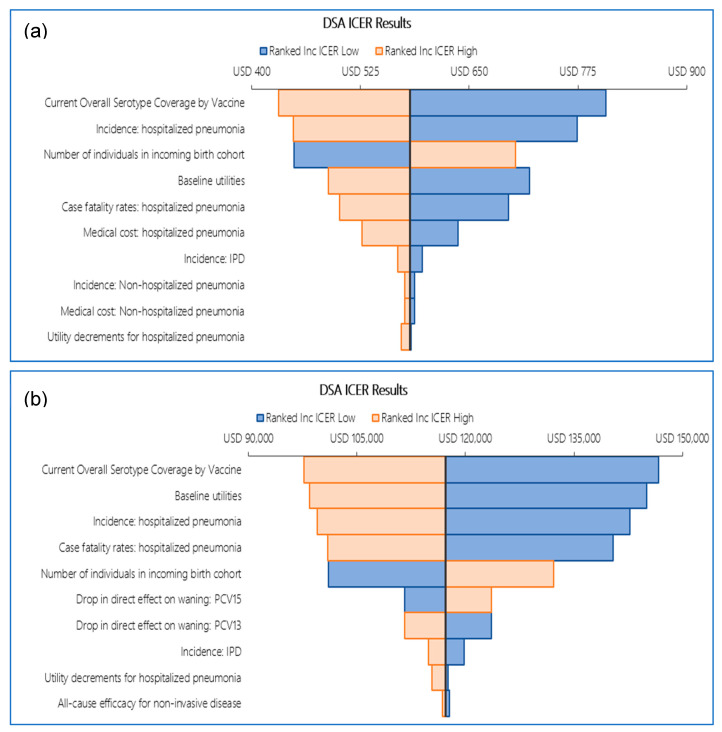

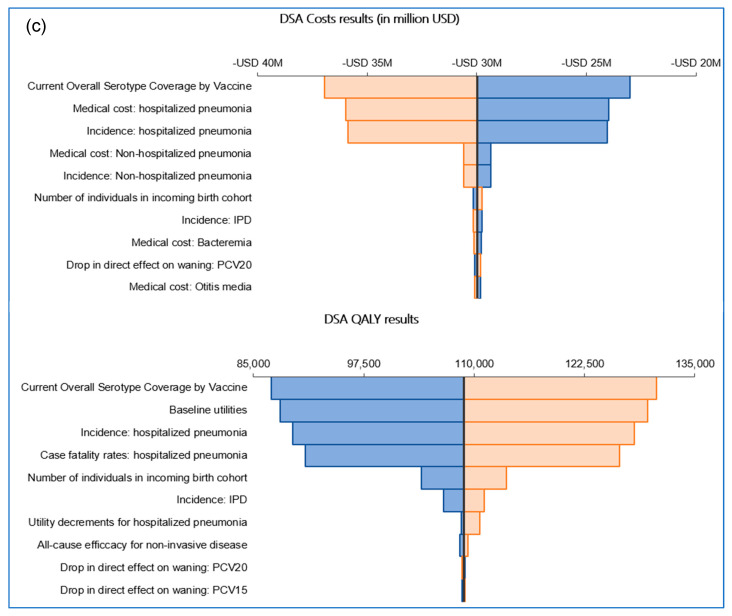
DSA tornado diagram reference case 2: (**a**) PCV13 vs. PCV10 (ICER), (**b**) PCV15 vs. PCV13 (ICER), (**c**) PCV20 vs. PCV15 (cost and QALYs). Abbreviations: DSA, deterministic sensitivity analysis; ICER, incremental cost-effectiveness ratio; inc, incremental; IPD, invasive pneumococcal disease; PCV10, 10-valent pneumococcal conjugate vaccine; PCV13, 13-valent pneumococcal conjugate vaccine; PCV15, 15-valent pneumococcal conjugate vaccine; PCV20, 20-valent pneumococcal conjugate vaccine; QALY, quality-adjusted life year.

In both reference cases, the DSAs showed similar results. In reference case 1, the comparisons of PCV13 versus PCV10 and PCV20 versus PCV15 were mostly influenced by vaccine serotype coverage, baseline utilities, hospitalized pneumonia incidence, pneumonia case fatality rate, and the vaccines’ all-cause efficacy against non-invasive disease. For the PCV15 versus PCV13 comparison, the most impactful parameters were the decrease in direct effect on waning for both vaccines, vaccine serotype coverage, baseline utilities, and hospitalized pneumonia incidence (Figure 3). For reference case 2 DSA, ICERs were computed for PCV13 versus PCV10 and PCV15 versus PCV13 comparisons when individual parameters were varied at their lower and upper bounds. The DSA identified vaccine serotype coverage, baseline utilities, hospitalized pneumonia incidence, pneumonia case fatality rate, and the number of newborns each year as the top ICER drivers for both comparisons (i.e., PCV13 versus PCV10 and PCV15 versus PCV13; see Figure 4).

For the pairwise comparison between PCV20 and PCV15, the variation in individual inputs influenced the magnitude of incremental costs, incremental QALYs, and ICERs, but it did not alter the relative ranking of vaccine strategies. Across all plausible ranges tested, PCV20 remained dominant, indicating that the finding is robust to uncertainty in the most influential model inputs. Therefore, ICER was not calculated. Instead, the DSA was conducted based on incremental costs and incremental QALYs for this comparison. The results from the DSA implied that incremental costs were mostly impacted by vaccine serotype coverage, medical costs and incidence for hospitalized and non-hospitalized pneumonia, whereas incremental QALYs were mostly affected by vaccine serotype coverage, baseline utilities incidents of hospitalized pneumonia, the case fatality rates for hospitalized pneumonia and the number of individuals in the incoming birth cohort (Figure 4).

**Figure 5:**
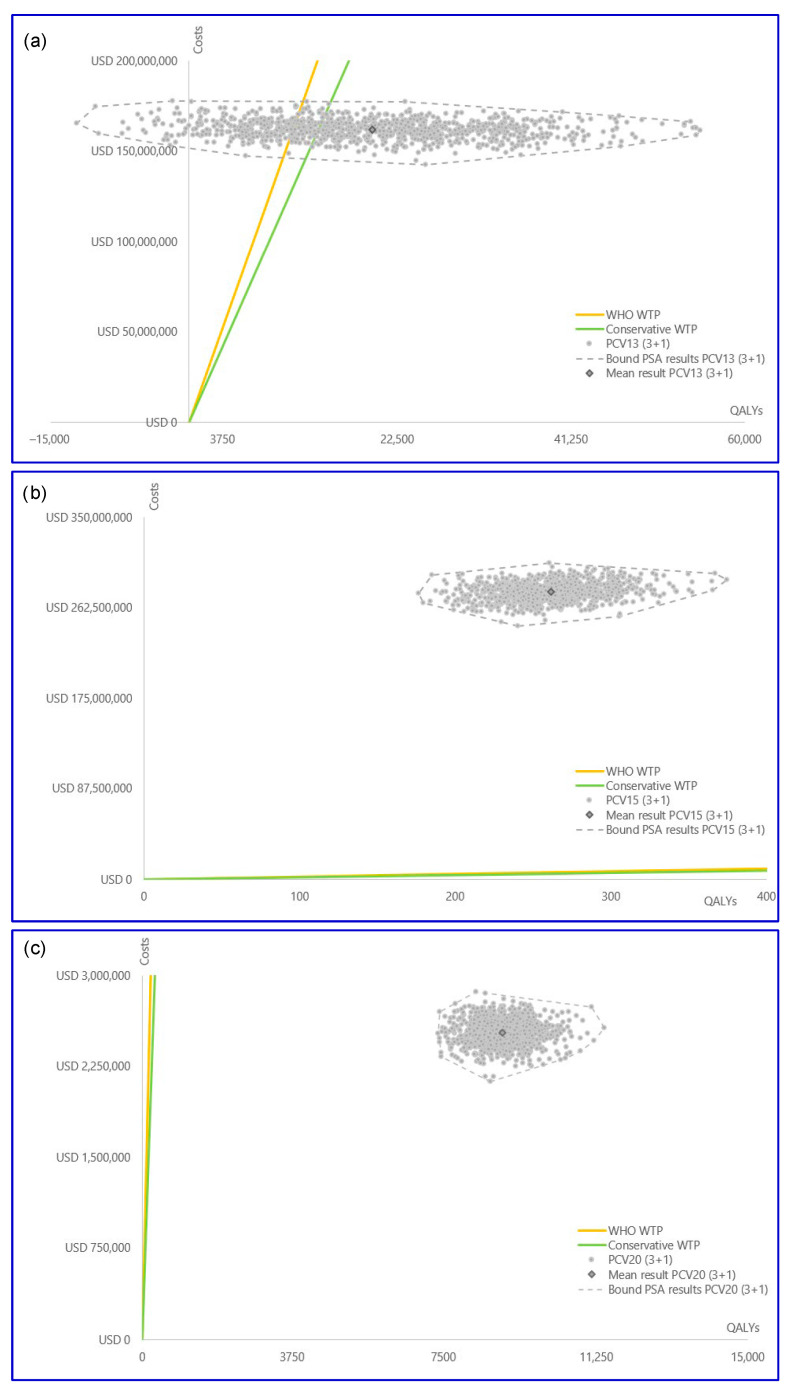
PSA scatterplots reference case 1: (**a**) PCV13 vs. PCV10, (**b**) PCV15 vs. PCV13, (**c**) PCV20 vs. PCV15. Costs are in USD. Abbreviations: PCV13, 13-valent pneumococcal conjugate vaccine; PCV15, 15-valent pneumococcal conjugate vaccine; PCV20, 20-valent pneumococcal conjugate vaccine; PSA, probabilistic sensitivity analysis; QALY, quality-adjusted life year; USD, United States dollar; WHO, World Health Organization; WTP, willingness-to-pay threshold.

**Figure 6:**
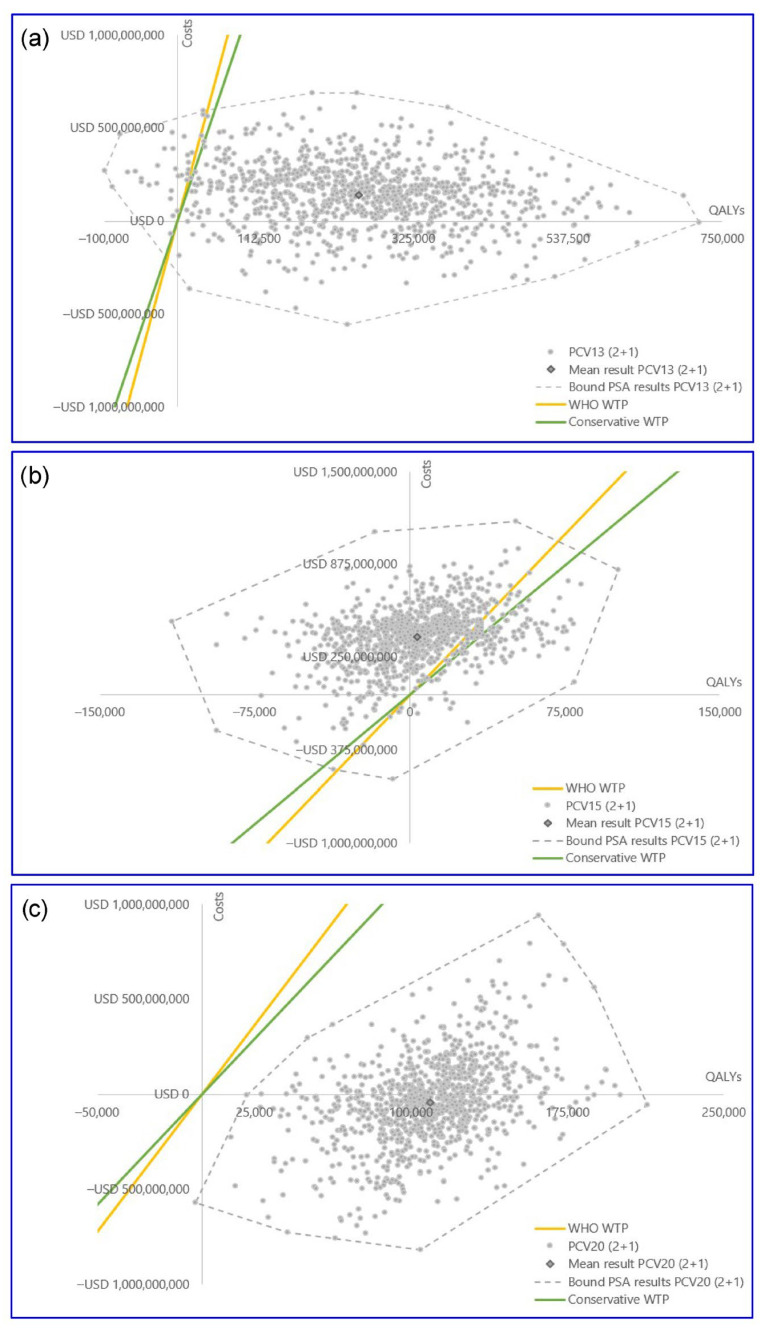
PSA scatterplots reference case 2: (**a**) PCV13 vs. PCV10, (**b**) PCV15 vs. PCV13, (**c**) PCV20 vs. PCV15. Costs are in USD. Abbreviations: PCV13, 13-valent pneumococcal conjugate vaccine; PCV15, 15-valent pneumococcal conjugate vaccine; PCV20, 20-valent pneumococcal conjugate vaccine; PSA, probabilistic sensitivity analysis; QALY, quality-adjusted life year; USD, United States dollar; WHO, World Health Organization; WTP, willingness-to-pay threshold.

The PSA results were consistent with the findings of both reference cases across all comparisons (Figure 5 and Figure 6). PCV13 generally outperformed PCV10 with a substantial proportion of simulations falling into the more effective quadrants and being cost-effective at both WTP thresholds. For the PCV15 versus PCV13 comparisons in reference case 1, PCV15 was more effective but was not cost-effective for both WTP thresholds in all iterations (Supplementary Table S5). In reference case 2, PCV15 was less effective in 44% of iterations, while the majority of iterations were found to be not cost-effective for both WTP thresholds. PCV20 consistently outperformed PCV15, being more effective and cost-effective in both reference cases and being less costly in more than 60% of iterations in reference case 2. Cost-effectiveness acceptability curves for all the comparisons are depicted in Supplementary Figures S3 and S4. The increased variation in PSA results for reference case 2 can be explained by the indirect effect inclusion in the variable parameters (Supplementary Table S6).

The results from the societal perspective scenarios for both reference cases are reported in Supplementary Tables S7 and S8, which support the findings from the base case analyses. Overall, the ICER for each pairwise comparison was slightly reduced, at less than 10% of the base case values, confirming robust results and conclusion.

## 4. Discussion

This cost-effectiveness analysis comprehensively assessed currently available PCVs, including two recently approved options (i.e., PCV20 and PVC15), in Vietnamese children using a stepwise and pairwise comparison framework under two scenarios (reference cases): with and without the consideration of the vaccine’s indirect protection under the assumption of PCVs mainly administered in the private vaccination centers with relatively lower vaccination rate or PCVs included in an NIP with higher vaccination rates. Across both reference cases, PCV13 emerged as a cost-effective option compared with PCV10 among vaccines currently used in the Vietnamese private market. This result was primarily driven by the greater associated health benefits, which resulted in significant savings in medical expenditures regardless of whether PCV is included in the NIP. PCV15 demonstrated comparable health benefits to PCV13, but considering the higher vaccination cost, PCV15 was estimated to be not cost-effective compared to PCV13 in both reference cases. In contrast, PVC20 consistently demonstrated an advantage in both reference cases. In reference case 1, PCV20 was estimated to be a more effective and cost-effective option compared with PCV15. In reference case 2, PCV20 was dominant over PCV15 across the 10-year time horizon from a payer perspective. The findings indicated that PCV20 would potentially represent the most favorable higher-valent option for inclusion in Vietnam’s pediatric NIP.

The findings from the sensitivity analyses, including both DSA and PSA, were consistent with the deterministic results of both reference cases with minimal deviations.

The results of this study are aligned with published economic evaluations in other countries. For example, in countries without PCVs in the NIP, including China [59] and Egypt [60], PCV13 was consistently found to be a cost-effective option among the lower-valent PCVs such as PCV10. When considering PCVs in the NIP, similar results were observed in Bhutan [61], the Philippines [62], Malaysia [63], Hong Kong [63] and India [64]. However, the studies in the Philippines, Malaysia, and Hong Kong reported that PCV13 achieved better value for the money than PCV10, as it was predicted to be a cost-saving option [62,63]. In addition, PCV20 was estimated to be the most favorable vaccination strategy compared with its lower-valent alternative (e.g., PCV15) in many countries, especially those with NIP in the Asia Pacific [12,13], Europe [14,15,16], and North America [17]. Similar findings and conclusions were also discussed in two recently published systematic reviews and meta-analyses of pneumococcal vaccines in children by Syeed et al., 2023 [64] and Vo et al., 2024 [65], both of which found PCV13 as a highly cost-effective option, while the latter study concluded that replacing PCV13 with PCV20 would lead to more cost savings and a higher QALY gain than transitioning to PCV15.

Like any modeling study, this analysis was inherently dependent on the quality and representativeness of the input data. In the current study, although Vietnam-specific data were prioritized as model inputs, the model was subject to several limitations. In reference case 1 assuming no NIP, the study focused exclusively on the pediatric population expected to benefit directly from vaccination (i.e., did not account for herd effects) as the assumed vaccination rate was relatively low. This methodology is considered conservative, particularly in light of the robust and sustained herd effects observed with PCVs worldwide [66]. However, when PCV was assumed to be included in the pediatric NIP (reference case 2), an indirect effect was applied, which resulted in substantial health gains estimated for all vaccines. Nevertheless, the findings were largely consistent with those from reference case 1, leading to the same conclusions. The health and economic impacts of sequelae due to pneumococcal disease (e.g., neurological impairment after meningitis) were not considered in any analyses, which represented a conservative assumption and was likely to underestimate the disease burden and vaccination impact; for example, Do et al., 2025 reported that 82.1% of meningitis cases in children under 2 years old could develop sequelae [6]. In addition, the present analysis is subject to uncertainty arising from limited age-specific epidemiological data in Vietnam, particularly incidence rates for pneumonia, OM, IPD in adults, and meningitis share in IPD. In the absence of national surveillance data, selected parameters were informed by evidence from a neighboring country—Thailand, which shares similar geographic, climatic, and demographic characteristics. The DSA results identified the incidence of hospitalized pneumonia and IPD as among the most influential drivers of model outcomes across both reference cases. This highlights the importance of these parameters in shaping cost-effectiveness results and underscores the value of improved country-specific surveillance data to further refine future evaluations.

Vaccine costs for all PCVs were based on publicly available list prices, as tender prices and negotiated discounts in Vietnam, although published by the Ministry of Finance, require hospital-specific tender codes to access winning bid prices, and no consolidated national database exists. Therefore, comparator price verification was not feasible. As a result, the cost-effectiveness estimated for higher-valent versus lower-valent vaccines may be conservative until more transparent procurement data become available.

Disease case costs were based on 2006 data for children under 5 and 2016 data for the population older than 5 years. Although the costs were inflated to 2025 prices, this cannot fully reflect changes in treatment practices and medical resource use. Therefore, the disease costs saved by the vaccines are likely underestimated. The serotype distributions for IPD used in both reference-case analyses were based on studies that were conducted in limited geographies and might not be representative of the true distributions in Vietnam. Vaccine uptake was based on some assumptions, given that PCVs had not yet been incorporated into Vietnam’s pediatric NIP at the time of the analysis. However, two reference cases with 50% and 90% vaccine uptake rates were analyzed, aiming to explore the impact of having PCV in the NIP as well as how vaccine uptake may impact the expected health and economic impacts from routinely vaccinating children with PCV. Lastly, the model estimated the vaccine’s direct effect using data from PCV13 effectiveness studies and PCV7 clinical trials due to a lack of robust data for PCV10, PCV15, and PCV20. However, PCV7 data have been used in numerous cost-effectiveness studies in PCVs by adjusting the PCV7 efficacy parameters to align with local serotype coverage, thereby reflecting regional differences in vaccine effectiveness [12,13,14,15,16,17]. Therefore, this was regarded as a reasonable method for estimating the direct impact of PCVs.

## 5. Conclusions

This cost-effectiveness analysis demonstrated that among the PCV options currently used in Vietnam, infant vaccination with PCV13 would be cost-effective compared with PCV10 from a payer perspective in Vietnam over 10 years. Among the next-generation, higher-valent vaccines, PCV20 consistently performed favorably relative to PCV15 under the private market assumptions and was dominant under the NIP assumption, reflecting its broader serotype coverage and associated health gains. While PCV20 showed the greatest projected health benefits among the vaccines considered, these findings should be interpreted within the context of the model assumptions and available data. The results further indicated that a wide uptake of pneumococcal infant immunization would generate disproportionately higher health benefits and substantial direct medical cost savings.

As Vietnam prepares to introduce a PCV into its NIP, these findings support the potential value of higher-valent PCVs and provide valuable evidence to inform national policy decisions while acknowledging the uncertainty inherent in the estimations.

## Figures and Tables

**Table 1:** Key inputs.

	Age Group, Years									
	<1	1	2	3	4	5–17	18–34 ^†^	35–49 ^†^	50–64 ^†^	≥65 ^†^
Population size at the start of the model (2025), n (millions) [24]	1.323	1.348	1.377	1.409	1.443	20.915	24.026	23.349	16.761	9.646
Disease incidence, cases per 100,000 individuals										
Invasive pneumococcal disease [25,26]	193.40	49.30	24.50	13.50	10.00	1.80	2.12	2.19	5.69	15.68
Hospitalized pneumonia [26]	3330.30	2897.10	2463.90	2030.70	1597.50	251.90	184.00	184.00	184.00	1849.72
Non-hospitalized pneumonia [26]	1435.30	1311.40	1187.40	1063.50	939.50	456.50	89.00	89.00	89.00	3825.61
Otitis media [26]	381.63	489.01	596.39	703.77	811.15	889.56	-	-	-	-
Proportion of invasive pneumococcal disease cases, %										
Meningitis [26]	10.29%	10.55%	10.90%	11.38%	12.12%	22.27%	12.20%	13.30%	19.14%	3.27%
Bacteremia [26]	89.71%	89.45%	89.10%	88.62%	87.88%	77.73%	87.80%	86.70%	80.86%	96.73%
Case fatality rate, %										
Meningitis [6,27]	8.80%	8.80%	8.80%	8.80%	8.80%	8.80%	20.80%	20.80%	20.80%	20.80%
Bacteremia [6,27]	8.80%	8.80%	8.80%	8.80%	8.80%	8.80%	20.80%	20.80%	20.80%	20.80%
Hospitalized pneumonia [26,28]	1.42%	1.42%	1.42%	1.42%	1.42%	1.42%	9.80%	9.80%	9.80%	9.80%
Direct medical costs (per episode) *, USD ($)										
Meningitis [29,30,31,32]	347.60	316.55	281.04	193.10	180.84	412.58	412.58	412.58	683.48	868.44
Bacteremia [29,30,31,32]	104.74	95.39	84.68	77.03	68.63	412.58	412.58	412.58	683.48	868.44
Hospitalized pneumonia [29,30,31,32]	91.26	83.10	73.78	67.12	59.80	412.58	412.58	412.58	683.48	868.44
Non-hospitalized pneumonia [29,30,31,32]	55.25	50.31	44.67	40.63	36.20	356.28	356.28	356.28	590.20	749.92
Otitis media [29,31,32]	45.55	41.48	36.83	33.50	29.84	29.84 ^‡^	-	-	-	-

* Direct medical costs of one case of disease for the 0- to 4-year-old groups were calculated based on Anh et al. [29] and inflated to 2025 prices using the CPI from the World Bank [31] and Trading Economics [32]. Direct medical costs of one case of disease for the ≥5-year-old groups were calculated based on Vo et al. [30] and inflated to 2025 prices using the CPI from the World Bank [31] and Trading Economics [32]. ^†^ Part of the model population only in reference case 2. ^‡^ Otitis for the 5 to 17-year-old group was assumed to be the same as for the 4-year-old group. Abbreviations: CPI, Consumer Price Index; USD, United States dollar.

**Table 2:** Vaccine direct and indirect effects: (**a**) direct effects; (**b**) indirect effect.

(a)																																									
							Source Data							Direct Vaccine Effect *																											
														PCV10							PCV13							PCV15							PCV20						
Hospitalized pneumonia [33]							25.50%							21.29%							24.71%							24.77%							26.29%						
Non-hospitalized pneumonia [34]							6.00%							5.01%							5.81%							5.83%							6.19%						
Otitis media [35]							7.80%							6.51%							7.56%							7.58%							8.04%						
IPD [36]							88.70%							59.67%							69.31%							69.43%							73.69%						
(**b**)																																									
						**Age Groups** **(Years)**						**Source Data (Unadjusted)**						**Indirect Vaccine Effect—Maximum Reduction**																							
																		**PCV10**						**PCV13**						**PCV15**						**PCV20**					
IPD [18,37]						<18						83.0%						55.83%						64.85%						64.97%						68.95%					
						18–49						88.0%						59.19%						68.76%						68.88%						73.11%					
						50–64						77.0%						51.80%						60.17%						60.27%						63.97%					
						≥65						73.0%						49.11%						57.04%						57.14%						60.65%					
Hospitalized pneumonia ‡ [38,39]						<5						30.5%						29.43%						34.19%						34.25%						36.35%					
						5–17						30.5%						23.93%						27.79%						27.84%						29.55%					
						18–49						15.0%						15.15%						17.60%						17.63%						18.71%					
						50–64						15.0%						16.92%						19.65%						19.69%						20.90%					
						≥65						15.0%						18.08%						21.00%						21.04%						22.33%					
Non-hospitalized pneumonia ‡ [38]						<5						22.5%						21.71%						25.22%						25.27%						26.81%					
						5–17						22.5%						17.65%						20.50%						20.54%						21.80%					
Otitis media ‡ [40]						<18						20.0%						18.83%						21.87%						21.91%						23.26%					
Indirect effect—Accrual over time ^┼^ [18,37]																																									
							Year 1							Year 2							Year 3							Year 4							Year 5–10						
All vaccines ^†^							37.5%							52.8%							67.7%							82.7%							100% ^†^						

* In the first year, a <12-month effect modifier of 75% in reference case 1 (i.e., 3 + 1 schedule) and 67% in reference case 2 (i.e., 2 + 1 schedule) was used to adjust the direct effect of the priming series only, reflecting a reduced vaccine effect of non-complete schedule (i.e., before the booster dose). ^┼^ Inputs were calculated based on Ladhani et al., 2018 [37], comparing PCV13 minus PCV7 serotypes (excluding serotype 3) in the PCV7 period (2010) to post-PCV13 (2011–2017). Year 6 of the PCV13 infant program was chosen as the steady-state year per Perdrizet et al., 2023 [18]. ^†^ The value of 100% indicates that the maximum incidence reductions were achieved, and a steady state was established. ‡ Indirect effects against non-invasive disease were adjusted using serotype distribution pre-PCV13. For hospitalized and non-hospitalized pneumonia, estimates for children were adjusted using serotype distribution pre-PCV13 at 70% and 86% for <2 years and 2–17 years [41], respectively, and at 67%, 60% and 56% for 18–49, 50–65, and ≥65, in turn [37]. Indirect effects against otitis media were assumed only in children < 18 years and adjusted using serotype distribution at 86% [37]. Abbreviations: IPD, invasive pneumococcal disease; PCV10, 10-valent pneumococcal conjugate vaccine; PCV13, 13-valent pneumococcal conjugate vaccine; PCV15, 15-valent pneumococcal conjugate vaccine; PCV20, 20-valent pneumococcal conjugate vaccine.

**Table 3:** Baseline utilities (using EQ-5D) and utility decrements.

General Population Utilities							
Age Groups, Years		0–24 *	25–34	35–44	45–54	55–64	≥65
Baseline utilities, males ^†^ [42]		0.92	0.94	0.91	0.90	0.89	0.84
Baseline utilities, females ^†^ [42]		0.92	0.92	0.90	0.90	0.87	0.83
Utility decrements [43,44,45,46,47]							
Age groups, years		0–17			≥18		
IPD	Meningitis	0.023			0.130		
	Bacteremia	0.008			0.130		
Hospitalized pneumonia		0.006			0.130		
Non-hospitalized pneumonia		0.004			0.045		
Otitis media		0.005			-		

* Utility values for the 0- to 14-year-old group were assumed to be the same as for 15- to 24-year-old group. ^†^ Share of female population was calculated based on World Bank data [24]. Abbreviation: IPD, invasive pneumococcal disease.

**Table 4:** Reference case 1 incremental results.

	Incremental Results		
	PCV13 vs. PCV10	PCV15 vs. PCV13	PCV20 vs. PCV15
Health outcomes			
Total pneumococcal disease cases *	−30,760	−399	−13,587
IPD cases	−1450	−19	−641
IPD—meningitis	−155	−2	−68
IPD—bacteremia	−1295	−17	−572
Hospitalized pneumonia cases	−23,570	−305	−10,411
Non-hospitalized pneumonia cases	−2979	−39	−1316
Otitis media cases	−2760	−36	−1219
Deaths due to pneumococcal disease	−453	−6	−200
QALYs	20,071	260	8865
Economic outcomes, USD			
Total cost	$162,114,546	$278,087,296	$2,529,090
Vaccination cost	$164,468,433	$278,117,801	$3,568,812
Total direct cost of disease	−$2,353,887	−$30,504	−$1,039,723
ICER, USD per QALY	$8077	$1,069,181	$285

* The total number of pneumococcal disease cases may vary from the sum of individual disease types due to rounding (i.e., integers are presented but the model estimates decimals, which are not presented here due to the binary nature of disease vs. non-disease states). Abbreviations: ICER, incremental cost-effective ratio; IPD, invasive pneumococcal disease; PCV10, 10-valent pneumococcal conjugate vaccine; PCV13, 13-valent pneumococcal conjugate vaccine; PCV15, 15-valent pneumococcal conjugate vaccine; PCV20, 20-valent pneumococcal conjugate vaccine; QALY, quality-adjusted life year; USD, United States dollar.

**Table 5:** Reference case 2 incremental results.

	PCV13 vs. PCV10	PCV15 vs. PCV13	PCV20 vs. PCV15
Health outcomes			
Total pneumococcal disease cases *	−298,240	−3865	−131,745
IPD cases	−6705	−87	−2962
IPD—meningitis	−708	−9	−313
IPD—bacteremia	−5998	−78	−2649
Hospitalized pneumonia cases	−189,229	−2452	−83,593
Non-hospitalized pneumonia cases	−45,846	−594	−20,251
Otitis media cases	−56,459	−732	−24,939
Deaths due to pneumococcal disease	−10,365	−134	−4579
QALYs	246,353	3193	108,823
Economic outcomes, USD			
Total cost	$143,291,680	$374,476,026	−$29,973,168
Vaccination cost	$222,069,266	$375,497,018	$4,828,541
Total direct cost of disease	−$78,777,586	−$1,020,992	−$34,801,709
ICER, USD per QALY	$582	$117,292	Dominant

* The total number of pneumococcal disease cases may vary from the sum of individual disease types due to rounding (i.e., integers are presented but the model estimates decimals, which are not presented here due to the binary nature of disease versus non-disease states). Abbreviations: ICER, incremental cost-effective ratio; IPD, invasive pneumococcal disease; PCV10, 10-valent pneumococcal conjugate vaccine; PCV13, 13-valent pneumococcal conjugate vaccine; PCV15, 15-valent pneumococcal conjugate vaccine; PCV20, 20-valent pneumococcal conjugate vaccine; QALY, quality-adjusted life year; USD, United States dollar.

## Data Availability

Data are contained within the article.

## Supplementary Materials

The following supporting information can be downloaded at https://www.mdpi.com/article/10.3390/vaccines14030220/s1, Figure S1: Reference case 1. Efficiency frontier for all the comparators combined; Figure S2. Reference case 2. Efficiency frontier for all the comparators combined; Figure S3. Cost effectiveness acceptability curves, reference case 1: (a) PCV13 vs PCV10, (b) PCV15 vs PCV13, (c) PCV20 vs PCV15; Figure S4. Cost effectiveness acceptability curves, reference case 2: (a) PCV13 vs PCV10, (b) PCV15 vs PCV13, (c) PCV20 vs PCV15; Table S1: Reference cases assumptions summary; Table S2. Serotype coverages of IPD reviewed from different sources; Table S3. Reference case 1—non-comparative results; Table S4. Reference case 2—non-comparative results; Table S5. Reference case 1—PSA results; Table S6. Reference case 2—PSA results; Table S7. Reference case 1. Scenario 1—Societal perspective results; Table S8. Reference case 2. Scenario 1—Societal perspective results.
