# Supplementary material for: Cost-Effectiveness of Infant Pneumococcal Conjugate Vaccination Strategies in Vietnam: A Stepwise Economic Evaluation
Source: Vaccines (Basel). 2026 Feb 27;14(3):220. doi: 10.3390/vaccines14030220 (PMC13030058; doi:10.3390/vaccines14030220)
Supplement: Supplementary file 1 [file vaccines-14-00220-s001.zip › vaccines-4103943-supplementary.pdf]

Supplementary Materials

Supplementary Table S1. Reference cases assumptions summary

	Reference case 1	Reference case 2
Model population	Pediatric population (<18 years old at the start of the model); each year newborns are added to the population.	Entire population of Vietnam; each year newborns are added to the population.
Vaccine target population	Children <2 years.	
Vaccination schedule and coverage	50% of the target population. Pneumococcal vaccine is not included in NIP.<br>All vaccines are administered using a 3+1 schedule (administered at 2, 4, 6, and 12–15 months of age).	90% of the target population. Pneumococcal vaccine is included in NIP.<br>All vaccines are administered using a 2+1 schedule (administered at 2, 4 and 12-15 months of age).
Comparisons	PCV10 vs PCV13; PCV13 vs PCV15; PCV15 vs PCV20, with a no-vaccination strategy as standard of care.	PCV10 vs PCV13; PCV13 vs PCV15; PCV15 vs PCV20, with a no-vaccination strategy as standard of care.
Direct effect	Assumed to be 0.75% of its maximum during the first year of life (as 3 out of 4 doses are administered). From year 2 (after the booster dose) until year 5, vaccine effect is at its maximum. From year 6 onward, vaccine effect is assumed to wane at 10% per year.	
Indirect effect (herd immunity)	No indirect effect is observed.	Unvaccinated population of all ages benefit from indirect effect, which is assumed to be accumulated from year 1 until year 5 of the analysis, reaching its full effect at year 5 and staying at the same level until the end of the analysis.

Abbreviations: NIP, National Immunization Program; PCV10, 10-valent pneumococcal conjugate vaccine; PCV13, 13-valent pneumococcal conjugate vaccine; PCV15, 15-valent pneumococcal conjugate vaccine; PCV20, 20-valent pneumococcal conjugate vaccine.

Supplementary Table S2. Serotype coverages of IPD reviewed from different sources

No	Authors	Study design and location	Study period	Age	Included in the calculation	Serotypes coverage			
						PCV10	PCV13	PCV15	PCV20
1	Nguyen THA et al., 2024 [1]	A cross-sectional study conducted at Hai Phon Children's hospital	November 2022 to September 2023	<15 years	Yes	37.00%	49.10%	49.10%	64.40%
2	Nguyen DT et al., 2024 [2]	Prospective surveillance study at 3 sentinel hospital sites in Vietnam	2015 to 2018	<5 years	Yes	74.24%	80.35%	80.35%	82.53%
3	Do HT et al., 2025 [3]	A retrospective cross-sectional study conducted at the Vietnam National Children's Hospital in Hanoi	2019 to 2022	<18 years	Yes	64.08%	82.39%	83.10%	88.03%
4	Bui AS et al., 2022 [4]	A prospective study conducted at Nghe An Obstetrics and Pediatrics Hospital in Nghe An Province	November 2019 to March 2021	2–59 months	Yes	84.92%	94.44%	94.44%	96.03%
5	Hieu CT et al., 2023 [5]	A retrospective hospital-based surveillance study from 2 children's hospital in Ho Chi Minh City	2012 to 2021	<5 years	Yes	76.10%	84.40%	84.40%	84.40%
6	Nguyen TBL et al., 2023 [6]	National Institute of Hygiene and Epidemiology and the National Children's hospital	2015 to 2021	<5 years	No (serotypes 6A and 6B are not differentiated)	72.20%	77.90%	78.30%	79.60%
**Average (among studies #1–5):**						**67.30%**	**78.10%**	**78.30%**	**83.10%**

Abbreviations: IPD, invasive pneumococcal disease; no, number; PCV10, 10-valent pneumococcal conjugate vaccine; PCV13, 13-valent pneumococcal conjugate vaccine; PCV15, 15-valent pneumococcal conjugate vaccine; PCV20, 20-valent pneumococcal conjugate vaccine.

Supplementary Table S3. Reference case 1 – non-comparative results

	SoC: No vaccination	PCV10 (3+1)	PCV13 (3+1)	PCV15 (3+1)	PCV20 (3+1)
Health outcomes					
Total pneumococcal disease cases*	5,893,985	5,703,637	5,672,877	5,672,479	5,658,892
IPD cases	40,397	31,423	29,972	29,954	29,313
IPD – meningitis	4,653	3,694	3,539	3,537	3,469
IPD – bacteremia	35,745	27,728	26,433	26,416	25,844
Hospitalized pneumonia cases	2,068,132	1,922,276	1,898,706	1,898,400	1,887,990
Non-hospitalized pneumonia cases	1,655,649	1,637,213	1,634,234	1,634,196	1,632,880
Otitis media cases	2,129,806	2,112,725	2,109,965	2,109,929	2,108,710
Deaths due to pneumococcal disease	32,495	29,693	29,240	29,235	29,035
QALYs	646,542,690	684,195,164	646,686,969	646,687,229	646,696,094
Economic outcomes, USD					
Total cost	$664,014,794	$1,423,627,237	$1,585,741,783	$1,863,829,079	$1,866,358,169
Vaccination cost	$0	$774,178,828	$938,647,261	$1,216,765,061	$1,220,333,873
Total direct cost of disease	$664,014,794	$649,448,409	$647,094,522	$647,064,018	$646,024,296

*The total number of pneumococcal disease cases may vary from the sum of individual disease types due to rounding (i.e., integers are presented but the model estimates decimals, which are not presented here due to the binary nature of disease vs non-disease states).

Abbreviations: IPD, invasive pneumococcal disease; PCV10, 10-valent pneumococcal conjugate vaccine; PCV13, 13-valent pneumococcal conjugate vaccine; PCV15, 15-valent pneumococcal conjugate vaccine; PCV20, 20-valent pneumococcal conjugate vaccine; SoC, standard of care; QALY, quality-adjusted life year; USD, United States dollar.

Supplementary Table S4. Reference case 2 – non-comparative results

	SoC: No vaccination	PCV10 (2+1)	PCV13 (2+1)	PCV15 (2+1)	PCV20 (2+1)
Health outcomes					
Total pneumococcal disease cases*	15,170,495	13,325,655	13,027,415	13,023,550	12,891,805
IPD cases	81,534	40,053	33,347	33,260	30,298
IPD – meningitis	8,607	4,228	3,520	3,511	3,198
IPD – bacteremia	72,928	35,825	29,827	29,749	27,100
Hospitalized pneumonia cases	5,705,233	4,534,877	4,345,648	4,343,195	4,259,602
Non-hospitalized pneumonia cases	7,253,921	6,970,261	6,924,415	6,923,820	6,903,569
Otitis media cases	2,129,806	1,780,465	1,724,006	1,723,274	1,698,336
Deaths due to pneumococcal disease	375,673	311,582	301,217	301,083	296,504
QALYs	2,371,539,234	2,373,063,170	2,373,309,523	2,373,312,716	2,373,421,539
Economic outcomes, USD					
Total cost	$6,500,190,527	$7,058,291,338	$7,201,583,019	$7,576,059,045	$7,546,085,876
Vaccination cost	$0	$1,045,231,741	$1,267,301,007	$1,642,798,026	$1,647,626,567
Total direct cost of disease	$6,500,190,527	$6,013,059,597	$5,934,282,011	$5,933,261,019	$5,898,459,309

*The total number of pneumococcal disease cases may vary from the sum of individual disease types due to rounding (i.e., integers are presented but the model estimates decimals, which are not presented here due to the binary nature of disease vs non-disease states).

Abbreviations: IPD, invasive pneumococcal disease; PCV10, 10-valent pneumococcal conjugate vaccine; PCV13, 13-valent pneumococcal conjugate vaccine; PCV15, 15-valent pneumococcal conjugate vaccine; PCV20, 20-valent pneumococcal conjugate vaccine; SoC, standard of care; QALY, quality-adjusted life year; USD, United States dollar.

Supplementary Table S5. Reference case 1 – PSA results

	PCV13 vs PCV10	PCV15 vs PCV13	PCV20 vs PCV15
Incremental QALY results			
Base-case value	20,071	260	8,865
PSA mean	19,790	261	8,933
Incremental costs results, USD			
Base-case value	$162,114,546	$278,087,296	$2,529,090
PSA mean	$162,013,175	$277,811,858	$2,529,386
Proportions of iterations per quadrant			
More costly/more effective	96.4%	100.0%	100.0%
More costly/less effective	3.6%	0.0%	0.0%
Less costly/less effective	0.0%	0.0%	0.0%
Less costly/more effective	0.0%	0.0%	0.0%

Abbreviations: PCV10, 10-valent pneumococcal conjugate vaccine; PCV13, 13-valent pneumococcal conjugate vaccine; PCV15, 15-valent pneumococcal conjugate vaccine; PCV20, 20-valent pneumococcal conjugate vaccine; PSA, probabilistic sensitivity analysis; QALY, quality-adjusted life year; USD, United States dollar.

Supplementary Table S6. Reference case 2 – PSA results

	**PCV13 vs PCV10**	**PCV15 vs PCV13**	**PCV20 vs PCV15**
Incremental QALY results			
Base-case value	246,353	3,192	108,823
PSA mean	249,642	3,520	109,183
Incremental costs results, USD			
Base-case value	$143,291,680	$374,476,026	-$29,973,168
PSA mean	$141,661,051	$386,802,965	-$41,807,855
Proportions of iterations per quadrant			
More costly/more effective	80.40%	55.30%	39.60%
More costly/less effective	1.70%	40.60%	0.00%
Less costly/less effective	0.10%	3.40%	0.10%
Less costly/more effective	17.80%	0.70%	60.30%

Abbreviations: PCV10, 10-valent pneumococcal conjugate vaccine; PCV13, 13-valent pneumococcal conjugate vaccine; PCV15, 15-valent pneumococcal conjugate vaccine; PCV20, 20-valent pneumococcal conjugate vaccine; PSA, probabilistic sensitivity analysis; QALY, quality-adjusted life year; USD, United States dollar.

Supplementary Table S7. Reference case 1. Scenario 1 – Societal perspective results

	Incremental results		
	**PCV13 vs PCV10**	**PCV15 vs PCV13**	**PCV20 vs PCV15**
Health outcomes			
Total pneumococcal disease cases*	-30,760	-399	-13,587
IPD cases	-1,450	-19	-641
IPD – meningitis	-155	-2	-68
IPD – bacteremia	-1,295	-17	-572
Hospitalized pneumonia cases	-23,570	-305	-10,411
Non-hospitalized pneumonia cases	-2,979	-39	-1,316
OM cases	-2,760	-36	-1,219
Deaths due to pneumococcal disease	-453	-6	-200
QALYs	20,071	260	8,865
Economic outcomes, USD			
Total cost	$ 161,691,211	$ 278,081,810	$ 2,342,102
Vaccination cost	$ 164,468,433	$ 278,117,801	$ 3,568,812
Total societal cost of disease	$ -2,777,222	$ -35,990	$ -1,226,710
ICER, USD per QALY	$ 8,056	$ 1,069,160	$ 264

*The total number of pneumococcal disease cases may vary from the sum of individual disease types due to rounding (i.e., integers are presented but the model estimates decimals, which are not presented here due to the binary nature of disease vs non-disease states).

Abbreviations: ICER, incremental cost-effectiveness ratio; IPD, invasive pneumococcal disease; PCV10, 10-valent pneumococcal conjugate vaccine; PCV13, 13-valent pneumococcal conjugate vaccine; PCV15, 15-valent pneumococcal conjugate vaccine; PCV20, 20-valent pneumococcal conjugate vaccine; QALY, quality-adjusted life year; USD, United States dollar.

Supplementary Table S8. Reference case 2. Scenario 1 – Societal perspective results

	Incremental results		
	**PCV13 vs PCV10**	**PCV15 vs PCV13**	**PCV20 vs PCV15**
Health outcomes			
Total pneumococcal disease cases*	-298,240	-3,865	-131,745
IPD cases	-6,705	-87	-2,962
IPD – meningitis	-708	-9	-313
IPD – bacteremia	-5,998	-78	-2,649
Hospitalized pneumonia cases	-189,229	-2,452	-83,593
Non-hospitalized pneumonia cases	-45,846	-594	-20,251
OM cases	-56,459	-732	-24,939
Deaths due to pneumococcal disease	-10365	-134	-4579
QALYs	246,353	3,193	108,823
Economic outcomes, USD			
Total cost	$ 141,749,590	$ 374,456,041	$ -30,654,349
Vaccination cost	$ 222,069,266	$ 375,497,018	$ 4,828,541
Total societal cost of disease	$ -80,319,676	$ -1,040,977	$ -35,482,890
ICER, USD per QALY	$ 575	$ 117,286	Dominant

*The total number of pneumococcal disease cases may vary from the sum of individual disease types due to rounding (i.e., integers are presented but the model estimates decimals, which are not presented here due to the binary nature of disease vs non-disease states).

Abbreviations: ICER, incremental cost-effectiveness ratio; IPD, invasive pneumococcal disease; PCV10, 10-valent pneumococcal conjugate vaccine; PCV13, 13-valent pneumococcal conjugate vaccine; PCV15, 15-valent pneumococcal conjugate vaccine; PCV20, 20-valent pneumococcal conjugate vaccine; QALY, quality-adjusted life year; USD, United States dollar.

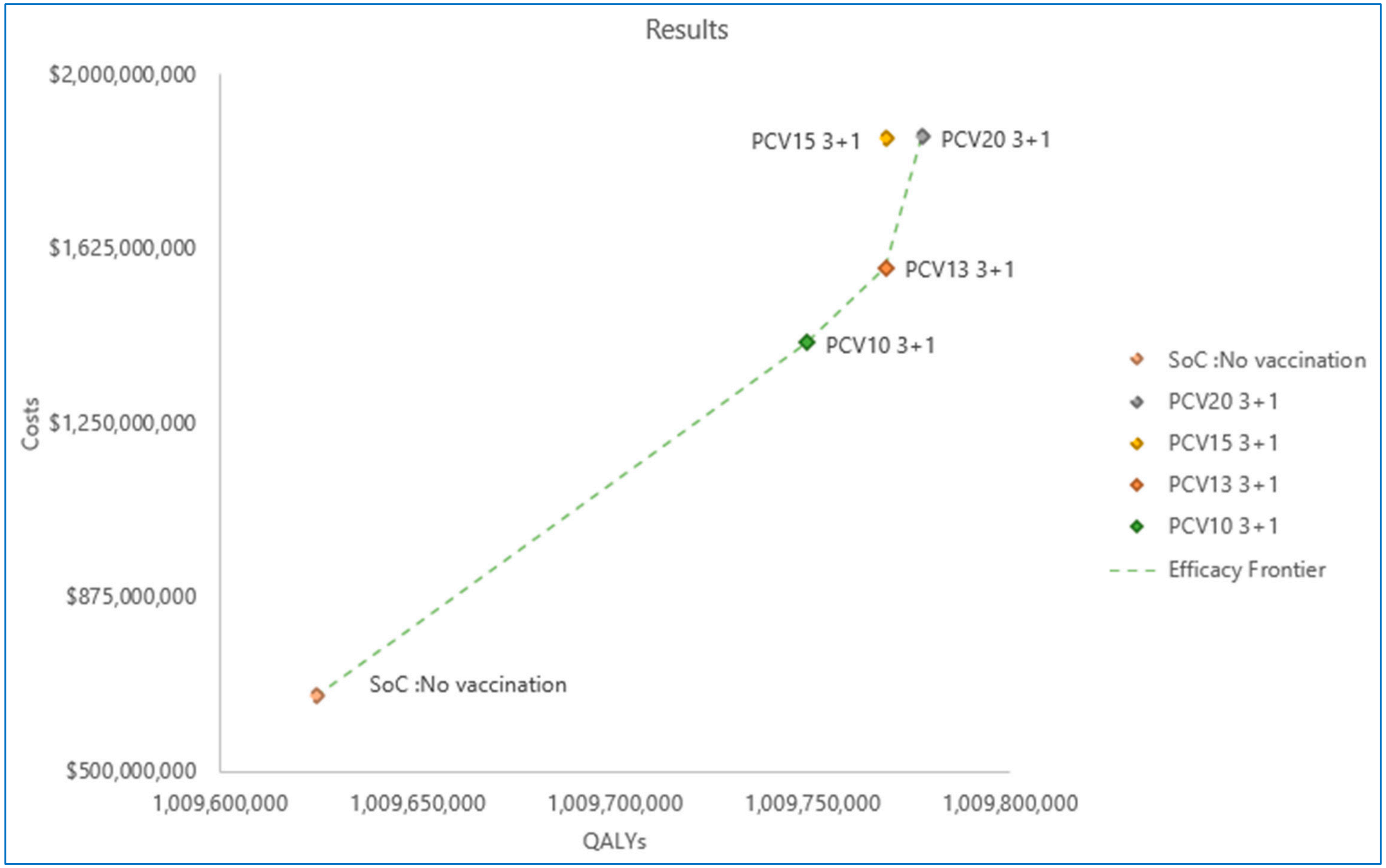

Supplementary Figure S1. Reference case 1. Efficiency frontier for all the comparators combined

Abbreviations: ICER, incremental cost-effectiveness ratio; IPD, invasive pneumococcal disease; PCV10, 10-valent pneumococcal conjugate vaccine; PCV13, 13-valent pneumococcal conjugate vaccine; PCV15, 15-valent pneumococcal conjugate vaccine; PCV20, 20-valent pneumococcal conjugate vaccine; QALY, quality-adjusted life year; SoC, standard of care

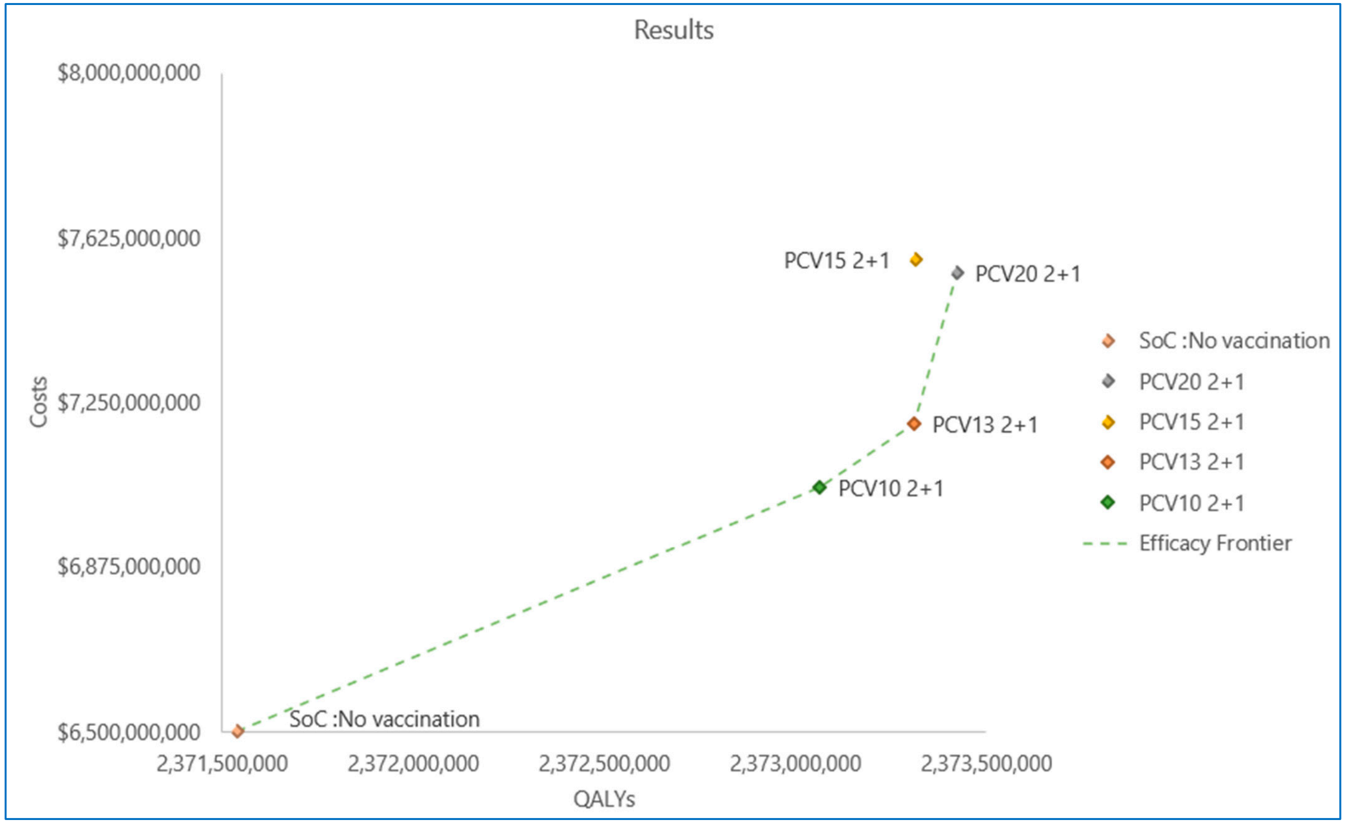

Supplementary Figure S2. Reference case 2. Efficiency frontier for all the comparators combined

Abbreviations: ICER, incremental cost-effectiveness ratio; IPD, invasive pneumococcal disease; PCV10, 10-valent pneumococcal conjugate vaccine; PCV13, 13-valent pneumococcal conjugate vaccine; PCV15, 15-valent pneumococcal conjugate vaccine; PCV20, 20-valent pneumococcal conjugate vaccine; QALY, quality-adjusted life year; SoC, standard of care

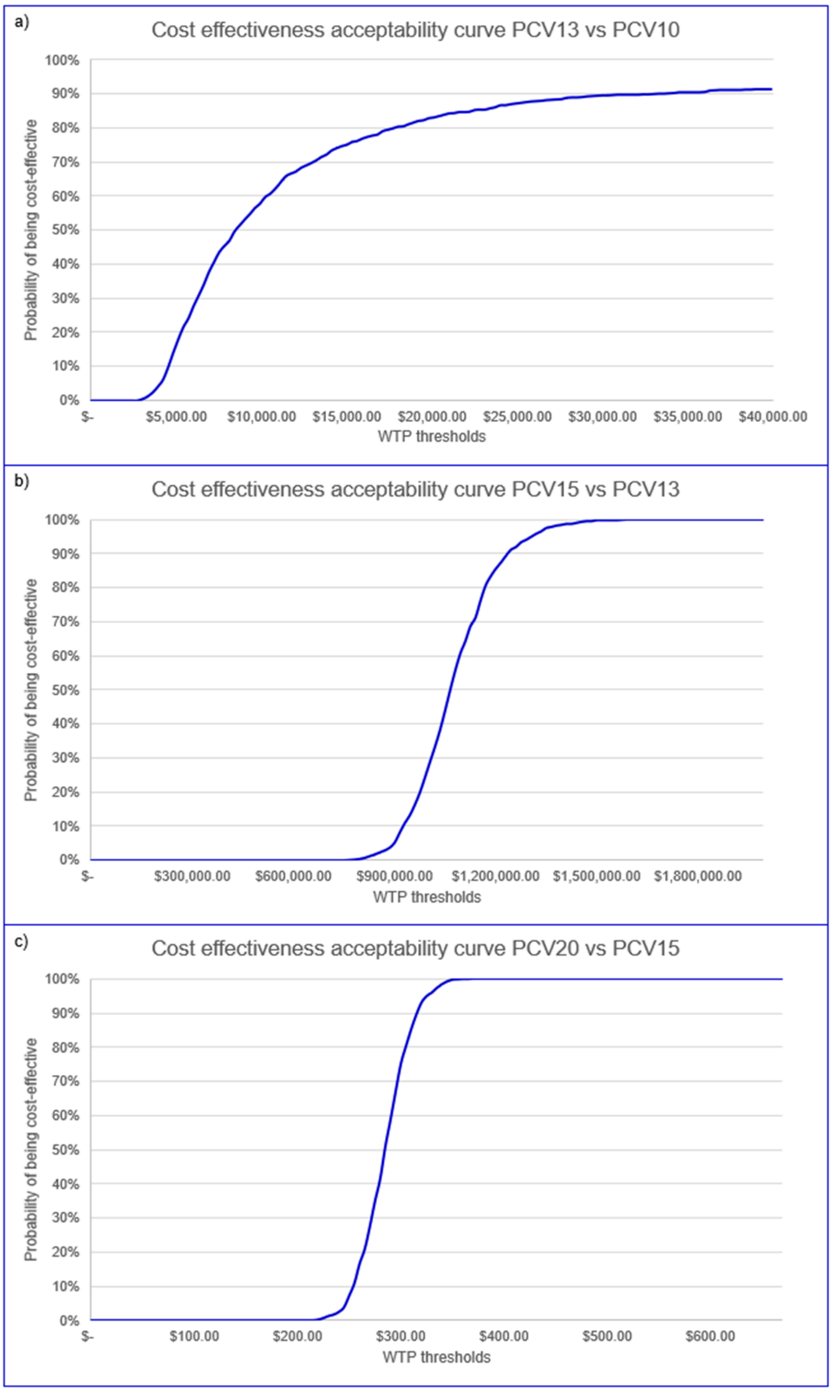

Supplementary Figure S3. Cost effectiveness acceptability curves, reference case 1: a) PCV13 vs PCV10, b) PCV15 vs PCV13, c) PCV20 vs PCV15

Abbreviations: PCV10, 10-valent pneumococcal conjugate vaccine; PCV13, 13-valent pneumococcal conjugate vaccine; PCV15, 15-valent pneumococcal conjugate vaccine; PCV20, 20-valent pneumococcal conjugate vaccine; WTP, willingness to pay.

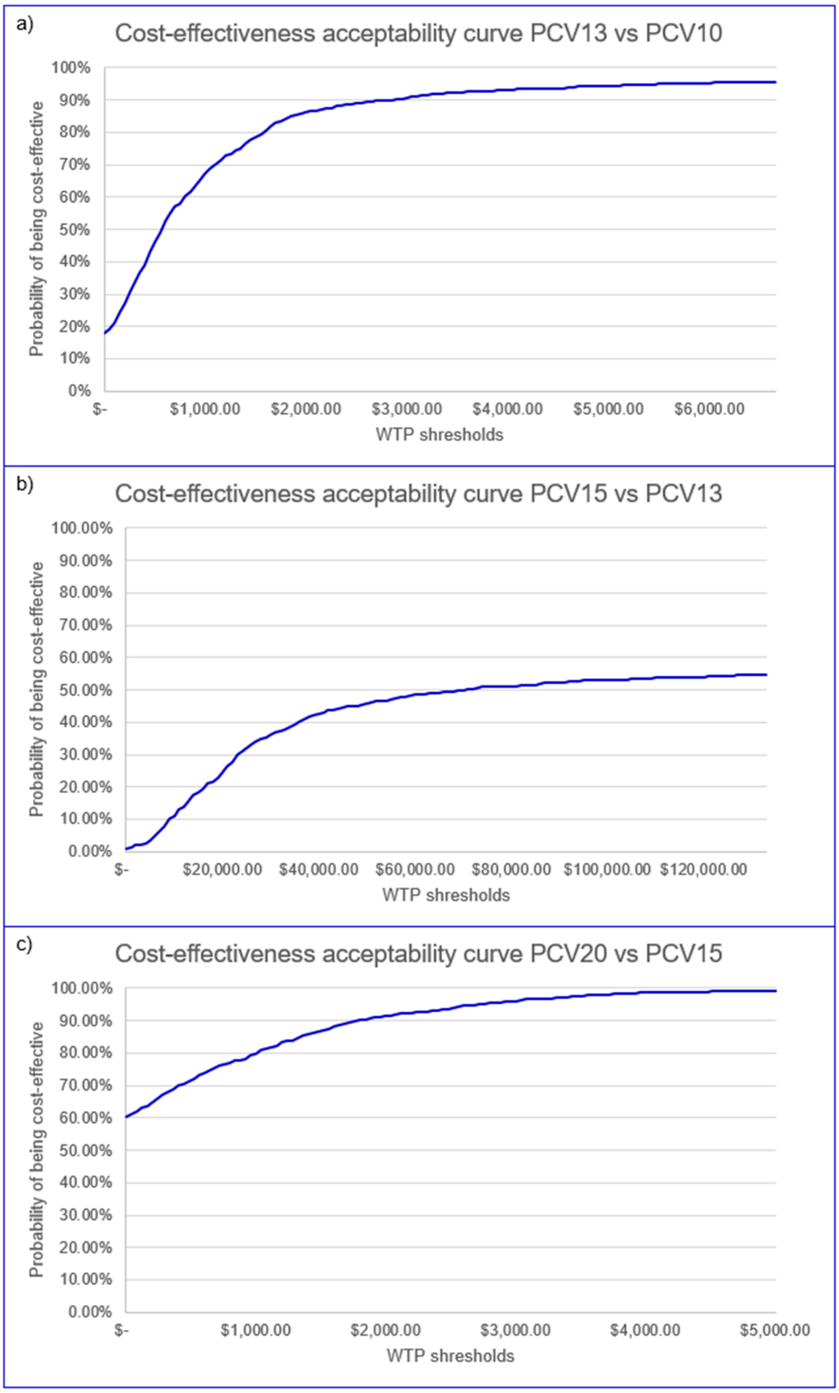

Supplementary Figure S4. Cost effectiveness acceptability curves, reference case 2: a) PCV13 vs PCV10, b) PCV15 vs PCV13, c) PCV20 vs PCV15

Abbreviations: PCV10, 10-valent pneumococcal conjugate vaccine; PCV13, 13-valent pneumococcal conjugate vaccine; PCV15, 15-valent pneumococcal conjugate vaccine; PCV20, 20-valent pneumococcal conjugate vaccine; WTP, willingness to pay.

References:

1. Anh, N.; Anh, L.; Phuong, T.; Anh, D.; Chien, B.; Hoa, T.; Tuyet, D.; Van, N.; Huy Hoang, L. Clinical characteristics of pediatric patients with pneumonia and Streptococcus pneumoniae serotype distribution at Hai Phong Children's Hospital, Vietnam. *Tạp chí Y học Dự phòng* **2024**, *T. 34 S. 4 (2024)*. DOI: 10.51403/0868-2836/2024/1744.
2. Nguyen, D. T.; Nguyen, T. L.; Olmsted, A.; Duong, T. H.; Hoang, H. M.; Nguyen, L. H.; Ouattara, M.; Milucky, J.; Lessa, F. C.; Vo, T. T. D.; et al. Epidemiology of pneumococcal meningitis in sentinel hospital surveillance of Viet Nam, 2015-2018. *BMC Infect Dis* **2024**, *24* (1), 1179. DOI: 10.1186/s12879-024-10065-0 From NLM.
3. Do, H. T.; Van Nguyen, L.; Nguyen, N. T. T.; Hoang, N. B. T.; Tran, D. M.; Nguyen, H. P. Serotype distribution of invasive Pneumococcal disease in a tertiary children's hospital in Vietnam. *BMC Infectious Diseases* **2025**, *25* (1), 412. DOI: 10.1186/s12879-025-10672-5.
4. Son, B.; Tang, X.; Cuong, T.; Chinh, D.; Le, T.-H.-H.; Dung, N.; Vu Nhat, D.; Anh, D. N. Serotype distribution and antibiotic resistance of Streptococcus pneumoniae isolates collected from unvaccinated children with pneumonia at a province in central Vietnam. *Iranian Journal of Microbiology* **2022**, *14*, 653–661. DOI: 10.18502/ijm.v14i5.10958.
5. Truong, H. C.; Van Phan, T.; Nguyen, H. T.; Truong, K. H.; Do, V. C.; Pham, N. N. M.; Ho, T. V.; Phan, T. T. Q.; Hoang, T. A.; Soetewey, A.; et al. Childhood Bacterial Meningitis Surveillance in Southern Vietnam: Trends and Vaccination Implications From 2012 to 2021. *Open Forum Infect Dis* **2023**, *10* (7), ofad229. DOI: 10.1093/ofid/ofad229 From NLM.
6. Liên, N. T. B.; Dương, T. N.; Anh, T. T. L.; Hương, N. T. T.; Đăng, L. H.; Trí, T. Q.; Khang, P. V.; Anh, N. T. H.; Anh, L. T. K.; Loan, N. T.; et al. Đặc điểm dịch tễ và phân týp huyết thanh các trường hợp viêm màng não do vi khuẩn Streptococcus pneumoniae ở trẻ dưới 5 tuổi điều trị tại bệnh viện Nhi Trung ương năm 2015 - 2021. *Tạp chí Y học Dự phòng* **2023**, *33* (2), 120–128. DOI: 10.51403/0868-2836/2023/1041 (accessed 2025/11/04).
